# Cast Clasps*Extract from an article printed in *Dental Items of Interest* for August, 1916.

**Published:** 1917-01

**Authors:** Norman B. Nesbett


### CAST CLASPS. *

BY NORMAN B. NESBETT.

Unfortunately, like many other things seemingly simple, the technique used in the construction is not easy and requires the utmost attention to detail and care in every step of the process.

The teeth to be clasped are cleaned and polished, and the occlusion noted, for space must be provided for the lugs at this time if it does not exist. Next an accurate plaster impression is taken, and this is the keynote of the whole process, for these cast clasps must have practically an inlay fit, and can not be made to fit by attempts at bending after construction.

---

* Extract from an article printed in *Dental Items of Interest* for August, 1916.

A small swivel crown-and-bridge tray is oiled, and the impression plaster used much thinner than for other impression work, for we wish to obtain sharpness and definition in all our tooth lines, and the stiffer mix of plaster will not flow into undercuts caused by overhanging teeth. The tray is removed, plaster scored at suitable points and removed in sections. Several impressions must be taken in some cases with long leaning teeth present before one accurate enough to be used can be obtained. A modeling compound impression of the opposing teeth, a small guide bite in wax, and shade of teeth are then secured. If this data is accurately obtained, the patient need not be seen again until the bridge is ready. The plaster impression is put together as soon as possible after taking, using the tray for a guide. Personally, I find it almost impossible to get the pieces in their proper places without some slight distortion unless I replace them in the tray and hold them in position with sticky wax. The teeth to be clasped are made removable, and as no grade of plaster is hard enough to do this work on, they are packed with either technic cement, Weinstein's artificial stone, or amalgam, sinking small dowels in the material before it begins to set. After setting the dowels are oiled and the rest of the impression poured in plaster. The compound impression is also poured and the two resulting models are mounted on an anatomical crown and bridge articulator, using the guide bite to establish occlusal relations. We now have a correctly articulated model with two removable teeth, having their dowel pins in the same plane so that one or both teeth can be removed freely. Inlay wax is then moulded to the teeth in the form we wish the clasps, and two castings made to represent the clasps. Casting is done on a Taggart oxyhydrogen machine and the Taggart technique followed throughout.

My first experiments in clasp casting were rather discouraging, for none of the existing alloys gave the spring or strength desired. Weinstein's formula, known as Ney Oro "B," gave better results. It was tough and strong, did not shrink so much, had a good color, but was finally abandoned as it lacked the spring desired. For the past six months I have been using a clasp metal, high in platinum content, having a melting point of 1960 degrees F., and requiring an oxyhydrogen flame for its successful melting and casting. A flame of less heat will melt the alloy so that it can be cast, but greater oxidization and slag formation takes place, resulting in a porous brittle casting that very much lacks the toughness and spring of the more quickly melted alloy. Furthermore, to insure an accurate fit, these clasps should be cast in a cool mould, and this can not be done where a prolonged heating is necessary to melt the alloy.

Recently I have been experimenting with a new alloy, platinum-white, or nearly so, in color, with a melting point approximately the same as pure gold, 1945 degrees F. This lower melting point is a distinct advantage, as it gives a smoother casting, with less chance of oxidization and resulting flaws in the casting. Like the high fusing clasp metal referred to before, this alloy has quite a complex formula. It is a combination of two of the new Weinstein formulas known as Elastic Gold and No. 68 Solder.

| | |
|---|---|
| Gold | 65.0 |
| Silver | 4.0 |
| Copper | 9.0 |
| Platinum | 8.5 |
| Palladium | 12.5 |
| Zinc | 1.0 |

After freeing the clasps from all adhering investment they are smoothed and fitted to the teeth on the model, and an Ash tube tooth ground so as to leave thickness enough for a saddle. I prefer the Ash tube teeth for this type of work, because they give me a maximum amount of porcelain and a minimum amount of solder, and when ground for correct occlusion they can be beatifully polished with no sacrifice of strength. The type of bicuspid and molars known as the Davis Crown, for metal work, can also be used to good advantage. The middle section, comprising the saddle, cup for porcelain and post, is then cast.

Usually I prefer to use for a post a high fusing clasp metal wire of No. 15 gauge (B. & S.), picked up in the casting and afterwards made doubly secure with a bit of solder. This middle section is cast with a platinum and palladium gold alloy known as Ney's casting gold "B"— formula for which is:

| | |
|---|---|
| Gold | 80.0 |
| Platinum | 9.6 |
| Palladium | 2.5 |
| Silver | 1.0 |
| Copper | 7.0 |

Melting point, 1,900 degrees F.

I prefer this alloy because it is untarnished by any oral secretions, does not appreciably change form in casting, and because 22K. or No. 84 solder can be used without fear of melting even thin edges. The middle section is placed in proper relation to the clasps on the model, plaster relations of the parts taken, invested and soldered. To secure the best results, the joints should be very close, so that only a minimum amount of solder will be required. The appliance is then tried on the model, the removable teeth making this easy, and any error in fit resulting from the soldering process corrected if necessary. If the clasps are as accurately fitted as they should be, any very slight change during the soldering will disturb the relations of the three sections so that they can not be placed in correct position, and the resulting appliance will not prove satisfactory.

*Under no circumstances* should the attempt be made to cast the two clasps and middle section in *one piece,* for while this can easily be done, no *proper fit can be obtained.* The warpage and shrinkage of such a large irregular casting is uncontrollable. The porcelain tooth is then cemented to place, all metal work nicely smoothed and polished and the appliance is again placed on the model and final adjustments for occlusion are made. This done, the ground surfaces of the porcelain are polished and the bridge is ready for the patient. If proper care and attention have been paid to each step, the bridge should require but little adjusting at the chair.

CONCLUSIONS.

Advantages—1st. No devitalization and usually no grinding of enamel surfaces necessary.

2nd. An appliance that can be easily removed and cleaned by any patient. Has no troublesome tubes, slots or sockets to be kept cleaned out as in other types of removable bridges.

3rd. Has a maximum of stability and food-grinding surfaces.

4th. Can be constructed with the minimum of nervous strain to the patient and the dentist.

5th. Is almost universal in application, as parallelism of abutments is not necessary.

Disadvantages—The disadvantages can be summed up under one head—that of liability of injury to the enamel surfaces under the clasps. We should select our cases for this appliance with great care, and not blind ourselves or our patients to the fact that dissolution of the enamel and caries of the dentin under *this* or *any other type of clasp will take place* unless strict attention to cleanliness is observed. The fact that this type of clasp does not allow room for any appreciable amount of food debris to crowd between it and the enamel should not lure us into a false sense of security when it is used on teeth of soft and chalky structure, or where the toilet of the mouth is neglected. I believe that with proper care and watchfulness on the dentists' and patients' part, time will show that no great amount of damage will occur under these bridges. And surely such small amount as may occur will be of less moment than the serious results that are coming from devitalized teeth.

